# Myocardial Function, Heart Failure and Arrhythmia in Marfan Syndrome: A Systematic Literature Review

**DOI:** 10.3390/diagnostics10100751

**Published:** 2020-09-25

**Authors:** Anthony Demolder, Yskert von Kodolitsch, Laura Muiño-Mosquera, Julie De Backer

**Affiliations:** 1 Centre for Medical Genetics, Ghent University Hospital, 9000 Ghent, Belgium; laura.muinomosquera@uzgent.be (L.M.-M.); julie.debacker@ugent.be (J.D.B.); 2 Department of Cardiology, University Heart Center, 20251 Hamburg, Germany; kodolitsch@uke.de; 3 Department of Paediatrics, Division of Paediatric Cardiology, Ghent University Hospital, 9000 Ghent, Belgium; 4 Department of Cardiology, Ghent University Hospital, 9000 Ghent, Belgium

**Keywords:** Marfan syndrome, heart failure, myocardial, ventricular arrhythmia

## Abstract

Marfan syndrome (MFS) is a heritable systemic connective tissue disease with important cardiovascular involvement, including aortic root dilatation and mitral valve prolapse. Life expectancy in patients with MFS is mainly determined by cardiovascular complications, among which aortic dissection or rupture are most dreaded. In recent years, heart failure and ventricular arrhythmia have drawn attention as extra-aortic cardiovascular manifestations and as additional reported causes of death. Imaging studies have provided data supporting a primary myocardial impairment in the absence of valvular disease or cardiovascular surgery, while studies using ambulatory ECG have demonstrated an increased susceptibility to ventricular arrhythmia. In this paper, current literature was reviewed in order to provide insights in characteristics, pathophysiology and evolution of myocardial function, heart failure and ventricular arrhythmia in MFS.

## 1. Introduction

Marfan syndrome (MFS) is a systemic connective tissue disease with autosomal dominant inheritance and a reported prevalence ranging from 1.5 to 17.2 per 100,000 individuals [1]. Cardiovascular, ocular and skeletal organ systems are most frequently involved in the Marfan phenotype. The most common clinical manifestations include aortic dilatation, mitral valve prolapse, lens luxation and skeletal abnormalities (disproportionally long limbs, scoliosis and pectus deformities). Other manifestations can be found in the integumental, pulmonary and central nervous organ systems. A wide phenotypic variability reflects the different extent to which various organ systems can be affected [1,2]. Diagnosis is based on the revised Ghent nosology, including aortic root dilatation and lens luxation as the two cardinal manifestations (Table 1) [2].

In the majority of patients, a (likely) pathogenic variant is found in the *FBN1* gene, encoding the extracellular matrix glycoprotein fibrillin-1, an important element in the assembly of microfibrils. Microfibrils may perform a structural role individually (in the extracellular matrix of elastic and non-elastic tissues), or unified as a supporting scaffold for elastin, thereby forming elastic fibers [3]. Elastic fibers play a central role in the structural integrity of connective tissues (e.g., in the aorta) by providing elasticity and tensile strength. In addition to the structural role, fibrillin-1 also plays a communicative role in biosignaling (regulating local bioavailability of TGF-β) and mechanosignaling (by interacting with mechanosensors and providing feedback to regulate the response to hemodynamic changes). Therefore, defects in fibrillin-1 may alter the structural integrity of connective tissue and may result in abnormal cellular signaling [3,4,5].

Life expectancy in patients with MFS is mainly determined by cardiovascular complications. Progressive dilatation of the proximal aorta is an important manifestation, rendering these patients at risk of aortic dissection or fatal rupture [6]. Although the aortic sinus is most commonly affected, aneurysms and dissections in more distal aortic regions and in extra-aortic arteries can also occur [7,8]. The reported prevalence of aortic root dilatation is slightly lower in children compared to adults (approx. 80% vs. 90%) [9,10]. Furthermore, data from the Genetically Triggered Thoracic Aortic Aneurysms and Cardiovascular Conditions (GenTAC) registry indicate that adult males are more likely than females to have aortic root dilatation (92% vs. 84%), aortic regurgitation (55% vs. 36%), and to have undergone prophylactic aortic root replacement (47% vs. 24%) [10]. Increased awareness, early detection, careful follow-up, life-style adjustments, pharmacological treatment and prophylactic surgery are currently established as the cornerstones of treatment in MFS. Implementation of these aspects in the treatment strategy has shown to substantially reduce the risk of type A dissection [6,11]. In patients with known (or suspected) MFS, echocardiography plays a central role in the identification, severity assessment and follow-up of cardiovascular abnormalities [6].

In recent years, heart failure and ventricular arrhythmia have drawn attention as additional cardiovascular manifestations of MFS [12]. Several imaging studies have provided data supporting a (sub)clinical, primary myocardial impairment in the absence of valvular disease or cardiovascular surgery in patients with MFS. In addition, studies using ambulatory ECG have demonstrated an increased susceptibility to ventricular arrhythmia [13,14]. These manifestations are also reflected in studies reporting on survival in patients with MFS, with heart failure and arrhythmia or sudden cardiac death (SCD) included as additional causes of death [14,15,16]. In this paper, we review current literature in order to provide insights in characteristics, pathophysiology and evolution of myocardial function, heart failure and ventricular arrhythmia in MFS.

## 2. Methods

**Figure 1:**
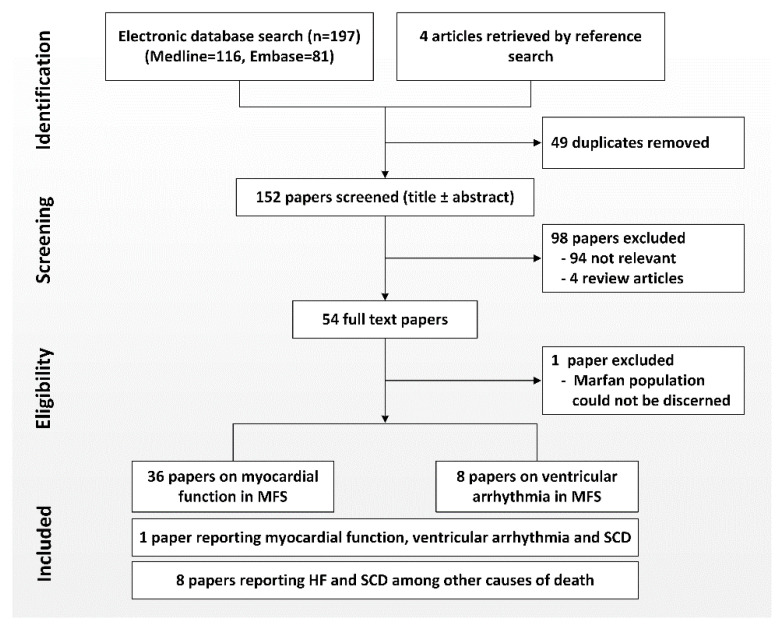
Flow chart of literature search. MFS: Marfan syndrome, SCD: Sudden Cardiac Death, HF: Heart failure.

We conducted a systematic review in accordance with the Preferred Reporting Items for Systematic Reviews and Meta-Analyses (PRISMA) statement to evaluate the current literature on myocardial function and arrhythmia in MFS [17]. Cohort studies, cross-sectional studies, case-control studies, case series and case reports were eligible for inclusion. First, a search of Medline and Embase was performed using the interchangeable search terms “Marfan syndrome”, “myocardial”, “ventricular”, “function”, “arrhythmia”, “heart failure” and “cardiomyopathy” in June 2020 (Figure 1). Next, a search in PubMed was performed to identify literature published ahead of print using the same search terms. Additional references were sought by examining citations in papers obtained through the specific searches. After deduplication, 154 papers were screened based on the title or abstract. A total of 56 full text papers were eligible for inclusion. Two studies included other genetic connective tissue disorders and were excluded since the population with MFS could not be discerned. The final selection consisted of 35 publications on myocardial function, eight publications on arrhythmia, eight publications reporting heart failure and SCD among other causes of death and one publication on both myocardial function, ventricular arrhythmia and SCD. Of the 35 publications on myocardial function, 22 studied myocardial function clinically, nine were reports on heart transplantation in MFS (seven case reports, one case series and one survey), one reported on the incidence of dilated cardiomyopathy after cardiovascular surgery and four studies assessed myocardial function in murine models of MFS. Results from the literature search are presented in tables and figures. Extracted information included author, study design, year, studied population, assessment methods and findings in MFS. Results of the articles were grouped, narratively synthesized and integrated with other relevant publications.

## 3. Myocardial Involvement

### 3.1. Left Ventricular Dimensions and Function: Evidence Obtained from Echocardiographic Studies

The first mention of possible myocardial involvement in MFS can be found in a case-report by Fujiseki et al. in 1984 [18]. Since then, various independent research groups hypothesized that myocardial impairment could be part of the MFS phenotype. Results from the studies conducted are summarized in Table 2. Several studies were designed to investigate left ventricular (LV) dimensions and systolic function in patients with MFS using echocardiography, with almost all of these studies excluding patients with significant valvular disease or previous aortic surgery [14,19,20,21,22,23]. Although in some of the early studies assessing LV dimensions and LV function [19,20,21,22], myocardial involvement was not clearly evidenced, subsequent studies reported the presence of increased LV dimensions in 7% to 68% (depending on the definition and the cohort), with mildly impaired LV systolic function (fractional shortening (FS) < 30%) present in approx. 10% of the patients [14,23]. In addition, mild impairment of diastolic function was demonstrated in multiple echocardiographic and cardiac magnetic resonance imaging (CMR) studies in adults and children with MFS [20,24,25,26]. Based on the coexistence of decreased ventricular compliance and reduced active myocardial relaxation, it was hypothesized that the impaired diastolic properties are attributable to reduced elastic recoil due to underlying connective tissue alterations [13].

Discrepancies between some of the previously mentioned studies led to further debate on myocardial involvement in MFS. A small number of studies reported no differences in LV diameters or systolic function compared to controls. Furthermore, some follow-up studies failed to detect changes in LV function or dimensions over time while others identified a small, yet significant, subgroup of patients presenting increased LV dimensions and reduced LV function [13,14,19,20,21,22,23,25]. These discrepancies can be attributed to (i) the small subgroup of patients affected, (ii) the mild degree of impairment in almost all of these affected patients and (iii) the lack of a uniform definition regarding myocardial involvement. Moreover, subtle changes in LV function are more difficult to identify and differentiate when using conventional (2D and M-mode) echocardiography as compared to more sensitive and advanced imaging techniques.

### 3.2. Evidence Obtained from Advanced Imaging Techniques

Additional insights in the LV function and volumes were provided in subsequent studies using tissue Doppler imaging (TDI), CMR and strain imaging. In 2006, De Backer et al. assessed diastolic filling in a small case-control study using TDI and systolic function using CMR in patients with MFS [24]. Compared to age- and sex-matched controls, patients with MFS showed signs of mild LV contractile dysfunction as expressed by a reduced ejection fraction (EF) (53.5 ± 9.0% vs. 59.6 ± 6.7%, *p* = 0.009), an increased indexed end-systolic volume (36.0 ± 9.5 vs. 29.5 ± 6.7 mL/m^2^, *p* = 0.007), and reduced peak systolic velocities. Furthermore, impaired diastolic function was observed in MFS [24]. Soon after, these findings were confirmed by two larger case-control studies [26,27].

Three subsequent studies using CMR provided additional evidence for myocardial involvement, as demonstrated by the observation of a reduced LV EF in a subgroup of patients [28,29,30]. In 2010, Alpendurada et al. evaluated 68 patients with MFS without significant valvular disease or prior cardiovascular surgery [28]. In this study, 25% of the patients had reduced LV EF on CMR. The reduced LV EF found in patients with MFS was mild, being less than 10% below the 95% confidence interval (CI) for sex and age reference values in most of the cases. Only 2 patients (2.9%) were diagnosed with heart failure in this study [28]. The relatively high rate of reduced EF in MFS patients in this study, could be attributed to the proposed cut-off value for reduced LV EF (below the 95% CI for sex and age decile). No association was found between reduced LV EF and age, gender, indexed aortic dimensions, presence of mitral valve prolapse or valve regurgitation, providing additional evidence that the impairment in ventricular function is inherent to the underlying connective tissue abnormality in MFS. Similar findings were observed in the CMR studies by de Witte et al. and by Winther et al., confirming that the reduced LV EF, is mostly mild but might affect a subgroup of patients in a more severe way [29,30]. By extending the detection prowess of conventional echocardiography, studies utilizing strain and strain rate imaging to assess and quantify changes in global and regional contractile function have confirmed the findings obtained from CMR [30,31,32,33].

### 3.3. Involvement of the Right Ventricle and Atria

Most studies have focused on the LV, but right ventricular (RV) involvement in MFS has also been suggested [34,35]. In the study by Kiotsekoglou et al., significant differences were found in tricuspid annular plane systolic excursion (TAPSE), rate of pressure rise (dp/dt) and pulsed TDI early filling measurements obtained over the lateral tricuspid valve corner, indicating impairment of RV function. In addition, atrial involvement was evidenced by reduced contractile, reservoir, and conduit function parameters for both atria [34,35]. The involvement of RV function was confirmed in the aforementioned CMR study by Alpendurada et al., showing that 10.3% of the patients also had a reduced RV EF [28]. Similarly, in the study by de Witte et al., RV EF was reduced compared to healthy controls (51% ± 7% vs. 56% ± 4%, *p* < 0.005) [29]. In both these studies, LV EF and RV EF were found to be strongly correlated, but the RV appears to be less frequently affected, possibly due to the higher workload imposed on the LV [28,29]. 

## 4. Pathophysiology of Marfan Cardiomyopathy

### 4.1. Intrinsic vs. Stressor-Induced Problem

Although almost all of the aforementioned studies reported myocardial impairment in the absence of previous cardiac surgery or significant valvular disease, very few patients were diagnosed with clinical heart failure. In contrast, there are several reports on end-stage heart failure necessitating heart transplantation in patients with MFS (Table 3). In addition, heart failure is mentioned as one of the leading causes of death in MFS (see further) [15,16,40,41,42,43,44,45,46,47,48,49]. Whether myocardial impairment and development of heart failure in MFS is a primary intrinsic problem or a secondary, stressor-induced problem remains an unanswered question.

### 4.2. Valvular Disease, Surgery and Genotype-Phenotype Relation

One of the cardiac stressors which may contribute in the development of heart failure is regurgitant valvular disease, which is frequently encountered in MFS and may induce volume overload [50,51]. By the age of 30, more than half of the patients with MFS will have mitral valve regurgitation, with severe mitral valve regurgitation reported in up to 12% of the patients [51]. Aortic valve regurgitation attributed to the dilatation of the aortic valve annulus is observed in up to 1 in 3 adult patients [50]. Since the prevalence of valvular disease tends to increase with age, it is likely that some patients with MFS will face some form of chronic volume overload caused by aortic and/or mitral regurgitation, inducing enlargement of the LV end-diastolic and end-systolic volume which may not be adequately compensated in some patients [51].

An association between prior aortic or valvular surgery and the development of heart failure as long-term complication in patients with MFS has also been suggested [48,52,53,54]. In several case reports and one case series describing patients with MFS undergoing orthotopic heart transplantation, almost all patients had a history of prior aortic or valvular surgery (Table 3) [40,41,42,43,44,45,46,47,49]. Similarly, a study by Hetzer et al. on a cohort of 421 patients with MFS who had undergone cardiac surgery reported cardiomyopathy in 11.2%. Only a minority of them already showed evidence of cardiomyopathy before the procedure. Even though occurrence of cardiomyopathy appeared to be independent of the type of myocardial protection and duration of ischemia, this study suggests that the performance of cardiovascular surgery on its own plays a role in the development of cardiomyopathy in patients with MFS [48]. Although these data point towards a relationship between heart failure and prior aortic surgery, it is also possible that this association reflects a subgroup of patients demonstrating a more severe phenotype, including a more vulnerable myocardium.

A third potential cardiac stressor is decreased aortic distensibility, which may contribute to impairment of LV function by altering the hemodynamic load imposed on the LV [55]. Aortic elasticity has been shown to be decreased in patients with MFS [56,57,58,59]. The relation between aortic elasticity and LV function has been assessed in the study by de Witte et al. using CMR and in the study by Loeper et al. using echocardiography. Both studies reported that the observed impairment of LV function was independent of aortic stiffness (based on measurable derivatives of aortic elasticity) [29,39]. However, confirmation and longitudinal data on these findings are required. A decrease in aortic distensibility can also be observed after aortic surgery during which a Dacron tube is implanted [60]. The difference in compliance of the Dacron implant compared to the original aorta may be higher than the difference in aortic compliance between patients with MFS and healthy individuals. A Dacron implant could lead to a slight but significant increase in LV afterload and thereby result in long-term cardiac stress. This hypothesis is supported by data reported by Nollen et al., demonstrating significantly lower distensibility in the tube graft compared to ascending aortic distensibility in patients without aortic root replacement [60]. Combined with the presence of a primary impairment of myocardial function in some patients, this could contribute to the prevalence of heart failure observed during long-term follow-up in patients after aortic surgery. Therefore, based on current data, a modulating role of aortic root replacement seems plausible [61].

In addition to these hemodynamic factors, it is conceivable that intrinsic factors play a role, including gene-related factors. Several studies have already shown that the type of underlying *FBN1* gene variant has an influence on aortic outcome [62,63,64,65,66]. Patients harbouring variants predicted to result in haploinsufficiency (HI) of fibrillin-1 show a worse outcome than carriers of variants predicted to result in a dominant negative (DN) effect [67]. In the same vein, a genotype-phenotype relationship in the myocardium can be suspected. Two studies have indicated a genotype-phenotype relation between myocardial impairment and underlying *FBN1* gene variants [33,68]. Aalberts et al. have shown an association between the type of underlying pathogenic *FBN1* variant and the development of LV dilatation in MFS [68]. Patients carrying non-missense variants (predicted to result in HI) more often demonstrated LV dilatation than those carrying missense variants (predicted to result in DN effect) [68]. Similarly, in a smaller study by Rahman et al. using three-dimensional speckle tracking echocardiography, LV EF, global LV circumferential strain and global LV area strain were all significantly lower in patients with variants predicted to result in HI than in those variants predicted to result in DN effect (*p* < 0.05) [33]. Different types of *FBN1* gene variants may also have a different effect on aortic elasticity [67], thereby potentially further contributing to impairment of myocardial function, but this has not been studied yet. In the aforementioned studies by de Witte et al. and Loeper et al., the relation between aortic elasticity and predicted HI or DN effect of *FBN1* gene variants was not evaluated [29,39]. Future studies should assess this relationship as the field of genotype-phenotype correlations may hold valuable information with implications for personalized therapeutic approaches [67].

### 4.3. Evidence Obtained from Mouse Models

In the quest to unravel the pathophysiology underlying Marfan cardiomyopathy, mouse models have provided clues to possible underlying mechanisms and pathways [37,69,70,71]. The presence and extent of fibrillin networks in the LV has been evidenced in both human and mouse studies [72,73,74]. Findings reported by Steijns et al. indicate that in wild-type mice, fibrillin-1 is present in different regions of the myocardium, including the apex, mid-ventricles and the atria [75]. These findings suggest that the mechanism of fibrillin-1 deficiency most likely also underlies the reported atrial and biventricular involvement.

In the *Fbn1^C1039G/+^* mouse model studied by Campens et al., impairment of cardiac function and structure remained mild and subclinical, resembling the myocardial phenotype observed in patients with MFS. Histologic examination of the myocardium revealed upregulation of TGF-β-related pathways and consistent abnormalities of the microfibrillar network, implicating a role for microfibrils in the mechanical properties of the myocardium [37]. In the fibrillin-1 deficient *Fbn*1*^mgR/mgR^* mouse model Cook et al. demonstrated that abnormal mechanosignaling by cardiomyocytes resulting from a deficient extracellular matrix caused dilated cardiomyopathy. The authors suggested that fibrillin-1 is implicated in the physiological adaptation of the myocardium to an increased workload and that dilated cardiomyopathy is a primary manifestation of MFS mice [69].

Two studies using the *Fbn1^C1039G/+^* mouse model tested the hypothesis that either pressure or volume overload on an already susceptible heart could result in a more severe dilated cardiomyopathy [70,71]. Valvular regurgitation and transverse aortic constriction ligation were shown to provoke dilated cardiomyopathy, while wild type controls remained fully compensated [70,71]. Taken together, these studies demonstrate the role of fibrillin-1 contributing to the cardiac reserve of the LV in the setting of cardiac stress [70,71].

### 4.4. Proposed Hypothesis

**Figure 2:**
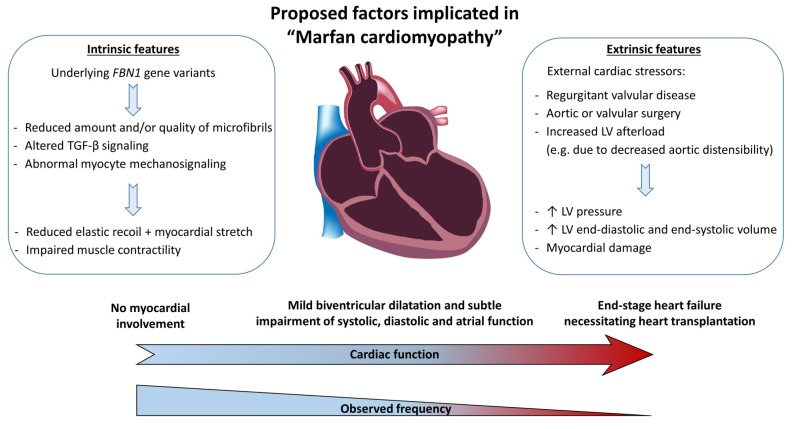
Proposed factors implicated in “Marfan cardiomyopathy”. FBN1: fibrillin-1 gene, TGF-β: transforming growth factor-beta, LV: Left ventricular.

Fibrillin-1 and microfibrils can be found throughout the myocardium as components of the extracellular matrix. They are assumed to play a role in sustaining proper cardiac function by contributing to the diastolic and systolic properties of the myocardium [72,73,74,75,76]. Underlying abnormalities in the *FBN1* gene are thought to result in abnormal mechanosignaling of the microfibrils which may cause inadequate compensation of cardiac stressors such as volume- or pressure overload in a subgroup of patients with MFS. Whether impaired elastic fiber function and/or impaired biosignaling is reflected in increased TGF-β signaling remains to be elucidated [37]. It is possible that a combination of intrinsic abnormalities (possibly variant-specific) renders the heart more vulnerable to cardiac stressors, resulting in an increased likelihood to develop myocardial dysfunction and ultimately heart failure (Figure 2).

## 5. Association with Arrhythmia

### Arrhythmia in Marfan Syndrome

In addition to aortic complications and cardiomyopathy, arrhythmia should be recognized as a relevant manifestation of the cardiac phenotype observed in MFS [14,52,54]. Several studies have associated MFS with an increased risk of arrhythmia, as summarized in Table 4. Studies based on data from ambulatory ECG in adults have demonstrated the presence of significant ventricular ectopy (defined as >10 premature ventricular contractions per hour) in 20–35% [14,77,78]. In children with MFS, the reported frequency of ventricular arrhythmia is much lower (7% demonstrating ventricular ectopy) [79]. Similarly, non-sustained ventricular tachycardia (NSVT) is reported in 10–20% of the adult patients with MFS and appears to be very rare in children [14,77,78,80]. However, ventricular tachycardia (VT) and SCD have been reported in both adults and children with MFS [14,81]. Four studies reported life-threatening arrhythmias in 7–9% of the patients and SCD, most likely due to arrhythmia, occurred in up to 4% [14,77,82,83]. Furthermore, fatal arrhythmias are reported in 12–19% of patients with MFS after aortic surgery, making it the 2nd most frequent cause of death in this setting [52,53,54].

The mechanisms underlying severe ventricular arrhythmia are multifactorial. Ventricular tachycardia and ventricular fibrillation (VF) usually arise from an initiating trigger in the presence of a proarrhythmogenic substrate (as observed in genetic channelopathies and cardiomyopathies), which allows the perpetuation of severe ventricular arrhythmias [84]. In patients with MFS, a proarrhythmogenic substrate may be present since subtle ECG changes have been identified independent of aortic root diameter, mitral and/or tricuspid valve prolapse or chamber dimension and function. Prolonged atrio-ventricular conduction time and altered depolarization is suggested by longer PQ- and QTc-intervals compared to healthy controls [80]. A (mildly) prolonged QTc-interval (>440 ms) has been described in 16–20% and 9–20% of adults and children respectively, while almost no patients present with QTc-intervals >500 ms [14,80,81,85]. The relevance of these subtle ECG changes remains understudied, but longer QTc-intervals have been associated with ventricular arrhythmia in MFS [14].

Patients with mitral valve prolapse, mitral valve regurgitation and previous aortic surgery have been reported to have higher risk of ventricular arrhythmia in several studies [14,52,53,54,77,78]. Furthermore, decreased LV EF, increased LV dimensions, prolonged QTc/QTu interval and high levels of N-terminal pro b-type natriuretic peptide (NTproBNP) have also been associated with ventricular ectopy independently of valvular disease and aortic surgery [14,77,82]. However, with the exception of NTproBNP, all these factors failed to predict severe arrhythmic events [77,82]. In addition, one study found an association between abnormal heart rate turbulence parameters and VT [83]. Genotype-phenotype correlations for arrhythmia have been performed in several studies, but only an association between mutations in exons 24–32 and VT/SCD has been found thus far [77,78,83]. These findings show that predictors of ventricular ectopy can be found, but the factors associated with severe arrhythmic events are more elusive, most likely because of the small number of patients experiencing severe arrhythmic events.

## 6. Heart Failure and Arrhythmia as Additional Causes of Death

**Figure 3:**
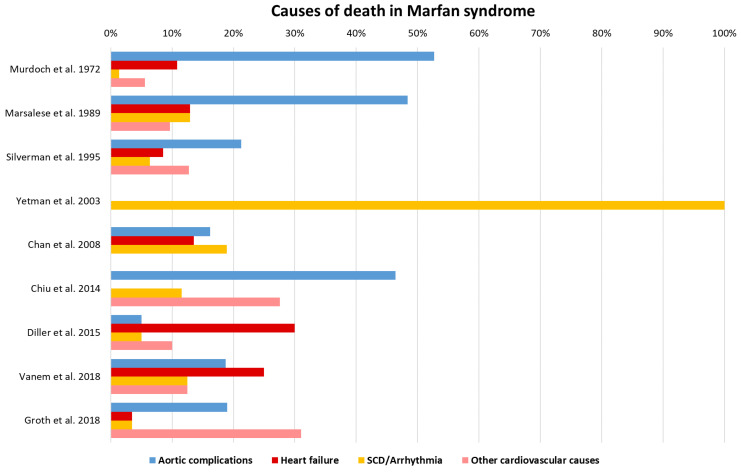
Overview of studies reporting causes of death in patients with MFS. SCD: Sudden cardiac death.

Most of the improvement in life expectancy achieved throughout the years in patients with MFS was obtained by focusing on prophylactic treatment and prevention of aortic events [16]. In 1995, Silverman et al. showed that the mean age at death of patients with MFS had significantly increased compared to the mean age at death in 1972 (41 ± 18 years versus 32 ± 16 years, *p* = 0.0023). Furthermore, patients undergoing aortic surgery after 1980 demonstrated even longer life expectancy [16]. Heart failure and arrhythmia as causes of mortality have been reported in later studies [14,15,16,86,87,88,89,90]. In Figure 3, cardiovascular causes of death in MFS are displayed as percentages of the total amount of deaths (non-cardiovascular causes of death were omitted).

As shown in Figure 3, the percentage of deaths attributed to heart failure and SCD (presumed arrhythmogenic) is approx. 30% and 20% respectively, with the exception of one study by Yetman et al. reporting SCD as the only cause of death in their patient cohort [15]. Although a wide variation in the reported numbers can be noted, it seems that, as the treatment and prevention strategies for aortic complications continue to improve, heart failure and arrhythmia constitute important cardiac manifestations requiring attention and awareness. When looking more specifically at post-operative survival in patients with MFS with prior aortic surgery, the three largest studies report heart failure and arrhythmia among the major causes of death [52,53,54]. Since these three studies were performed by the same research group, a significant overlap should be taken into account when considering these numbers.

In their most recent study in 2009, Cameron et al. reported results after aortic root replacement in 373 patients with MFS in a time period of more than 30 years [54]. In these three studies, dissection or rupture of the residual aorta remains the main cause of death, occurring in up to 19% of the patients. When looking at the extra-aortic causes of death, arrhythmia stands out, occurring in 12–19% of the patients and thereby rivaling aortic events as the leading cause of death. Heart failure on the other hand is reported as the cause of death in 2–10% of the patients, which appears to be less frequent compared to data from aforementioned studies reporting on survival in MFS.

## 7. Discussion

### 7.1. Current View on Marfan Cardiomyopathy

Taken together, the aforementioned studies confirm that ventricular dimensions as well as systolic and diastolic function are well within normal limits in the vast majority of patients with MFS. However, even in the absence of cardiac surgery or significant valvular disease, a mild biventricular dilatation with diastolic and systolic dysfunction in a subgroup of patients with MFS has been repeatedly reported [20,23,24,25,26,28,35]. Since myocardial involvement was reported in the absence of any cardiac surgery or significant valvular abnormalities, this phenotypic expression was designated an “intrinsic” or “inherent” dysfunction of the myocardium and was termed “Marfan cardiomyopathy”. Advanced imaging techniques (such as CMR, TDI, strain and strain rate imaging) appear to be more suited to detect these alterations. Despite these findings, almost no patients were diagnosed with clinical heart failure in the aforementioned studies. Follow-up studies to better identify those patients at risk of clinically relevant myocardial dysfunction are still required.

### 7.2. The Intertwined Mechanism of Marfan Cardiomyopathy and Ventricular Arrhythmia

The relation between a reduced amount or quality of extracellular fibrillin in the myocardium, a primary impairment of myocardial function, increased likelihood of ventricular ectopy and possible alterations in the electrophysiological substrate remains unclear. It is possible that, due to the reduced amount or quality of fibrillin, mechanical forces imposed on the cardiomyocytes in patients with MFS may be less adequately compensated than in healthy individuals. Therefore, chronic or acute myocardial dilatation and associated stretch could perhaps induce (complex) ventricular ectopy more easily in these patients where subclinical myocardial impairment is noted. In addition, the impairment of myocardial function observed in some patients may also signify inherent abnormalities in the underlying electrophysiological substrate. The combination of (complex) ventricular ectopy together with the alterations in electrical and/or mechanical properties of the heart may be severe enough to induce SCD in some patients with MFS, as suggested in studies by Hoffmann et al. [82] and by Yetman et al. [14]. Furthermore, increased NT-proBNP has been demonstrated as independent predictor of both diastolic dysfunction and severe arrhythmic events [38,82]. This may signify that long-term mild myocardial stretch potentially predisposes these patients to (severe) ventricular arrhythmia [38,82].

## 8. Current Limitations and Evidence Gaps

To date, large multicentre studies reporting the overall incidence or prevalence of heart failure and severe arrhythmia in MFS are lacking. Therefore, identification of predisposing factors is limited. Additional studies are necessary to evaluate the clinical relevance of Marfan cardiomyopathy and ventricular ectopy, to elucidate the underlying mechanisms in MFS and to allow better risk stratification of patients with MFS. Information on these aspects could hold important implications for developing strategies to treat heart failure and ventricular arrhythmia in MFS.

We should also take into account that—certainly in the case of older studies—some of the patients enrolled may have had some other form of Heritable Thoracic Aortic Disease, caused by pathogenic variants in genes other than *FBN1*. Advancing insight in recent years shows us that caution is advised in grouping all these conditions.

## 9. Conclusions

Myocardial involvement in the absence of valvular disease can be observed in patients with MFS, usually presenting as mild, asymptomatic impairment of LV systolic and diastolic function. In addition, some patients with MFS present (complex) ventricular arrhythmia as well as alterations in repolarization. A subgroup of patients with MFS tends to develop heart failure, severe arrhythmia and SCD, in which the effects of cardiac stressors may play an important role. Reduced myocardial function, heart failure and ventricular arrhythmia should be considered an essential concern of medical care for patients with MFS. Careful assessment of these features should be added to the standard aortic evaluation.

## Figures and Tables

**Table 1:** Revised Ghent criteria for diagnosis of MFS [2].

**In the Absence of Family History of MFS:**
(1) Ao * (Z-score ≥ 2) AND EL = MFS
(2) Ao * (Z-score ≥ 2) AND causal *FBN1* mutation = MFS
(3) Ao * (Z-score ≥ 2) AND systemic score ≥ 7 points = MFS
(4) EL AND causal *FBN1* mutation with known Ao = MFS
**In the Presence of Family History of MFS:**
(5) EL AND family history of MFS = MFS
(6) Systemic score ≥ 7 points AND family history of MFS = MFS
(7) Ao * (Z-score ≥ 2 above 20 years old, ≥ 3 below 20 years) + family history of MFS = MFS

* Ao = Aortic diameter at the sinuses of Valsalva above indicated Z-score or aortic root dissection. EL = ectopia lentis; MFS = Marfan syndrome.

**Table 2:** Summary of studies assessing myocardial function in MFS.

Author	Type of Study	Number of Patients with MFS	Controls	Assessment	Findings in MFS
Roman et al. 1989 [19]	Case-control	59 children and adults (51% female)	59 age- and sex-matched controls 59 age- and sex-matched subjects with MVP	M-mode 2D echo	Similar LV diameter and systolic function Increased LV mass
Savolainen et al. 1994 [20]	Case-control	22 children (64% female)	22 age-matched healthy children	M-mode Doppler CMR	Similar LV diameter and systolic function LV diastolic dysfunction
Porciani et al. 2002 [21]	Case-control	20 adult MFS and 8 MASS phenotype (54% female)	28 healthy, age and gender-matched controls	M-mode 2D echo Doppler	Similar LV diameter and systolic function LV diastolic dysfunction
Yetman et al. 2003 [14]	Follow-up: 6 years ^ (1.6–24.5)	70 children and adults (51% female)	/	2D echo	68% had LV dilatation (LVEDD Z-score > 2) 11% had LV systolic dysfunction (FS < 30%)
Chatrath et al. 2003 [22]	Follow-up: 10.8 years * (1–29)	36 children and adults (36% female)	/	M-mode	19% had LV dilatation (LVEDD > 95% above normal limits) No change in LV dimensions during follow-up No LV systolic dysfunction
Meijboom et al. 2005 [23]	Follow-up: 6 years * (0.3–15)	234 adults (51% female)	/	M-mode 2D echo	9% had mild LV dysfunction (FS 25–30%) 7% had LV dilatation (LVEDD > 117% (2SD + 5%)) 3% developed LV dilatation during follow-up 1% had LVEDD > 112% and FS < 30%
De Backer et al. 2006 [24]	Case-control	26 adults (54% female)	26 age and sex-matched controls	2D echo Doppler TDI CMR	LV systolic and diastolic dysfunction LV dilatation
Das et al. 2006 [25]	Case-control	40 children and adults (68% female)	40 age and sex-matched controls	M-mode Doppler	Similar systolic function LV diastolic dysfunction LV dilatation
Rybczynski et al. 2007 [26]	Case-control	55 adults (49% female)	86 healthy controls	2D echo Doppler TDI	LV diastolic dysfunction LV systolic dysfunction
Kiotsekoglou et al. 2008 [27]	Case-control	66 adults (44% female)	61 healthy controls	M-mode 2D echo Doppler TDI	17% had LV dilatation (predicted LVEDD ≥ 112% and FS ≥ 25%) LV diastolic dysfunction LV systolic dysfunction
Kiotsekoglou et al. 2009 [34]	Case-control	66 adults (44% female)	61 age, sex, height, weight, and BSA-matched normal volunteers	M-mode 2D echo Doppler TDI	RV systolic dysfunction RV dilatation Increased right atrium area
Kiotsekoglou et al. 2009 [35]	Case-control	72 adults (42% female)	73 age, sex, and BSA-matched controls	2D echo Doppler TDI	LV diastolic dysfunction RV diastolic dysfunction Atrial systolic and diastolic dysfunction
Alpendurada et al. 2010 [28]	Cross-sectional	68 adults (40% female)	/	CMR	25% had reduced LV EF (below 95% CI for sex and age decile) 10% had reduced RV EF (below 95% CI for sex and age decile) LV dilatation RV dilatation
Kiotsekoglou et al. 2011 [31]	Case-control	44 adults (41% female)	49 controls without significant differences in age, sex, height, weight, and BSA	M-mode 2D echo Doppler Strain rate imaging	20% had LV dilatation (predicted LVEDD ≥ 112% and FS ≥ 25%) LV diastolic dysfunction LV systolic dysfunction
de Witte et al. 2011 [29]	Case-control	144 adults (51% female)	19 healthy controls	CMR	9% had reduced LV EF (<45%) LV systolic dysfunction RV systolic dysfunction
Scherptong et al. 2011 [36]	Case-control Follow-up: 4 years ^	50 adults (50% female)	50 controls matched for age, sex, and BSA	M-mode 2D echo Doppler Strain rate imaging	Similar LV and RV EF LV systolic dysfunction RV systolic dysfunction No changes in systolic or diastolic function during follow-up
Angtuaco et al. 2012 [32]	Case-control	16 children and adults (56% female)	26 controls without significant differences in sex, race, age, weight, height, and BSA	M-mode 2D echo Doppler Strain rate imaging	LV systolic dysfunction No significant differences in strain
Abd El Rahman et al. 2015 [33]	Case-control	45 children and adults (42% female)	40 age-matched healthy controls	M-mode 2D and 3D echo Doppler 3D speckle tracking	LV diastolic dysfunction LV systolic dysfunction Left atrial diastolic dysfunction No differences in left atrial systolic function No differences in M-mode LVEDD, LVESD and FS
Campens et al. 2015 [37]	Case-control Follow-up: 6 years * (3.4–10.3)	19 adults (47% female)	19 age and sex-matched controls	2D echo Doppler TDI	No changes in LV dimensions during follow-up No changes in LV systolic or diastolic function during follow-up
Gehle et al. 2016 [38]	Case-control	217 children and adults (51% female)	339 patients referred for suspected MFS (diagnosis ruled out according to the Gh. nosology)	M-mode 2D echo Doppler TDI NT-proBNP	Increased NT-proBNP levels LV diastolic dysfunction LV dilatation No signs of LV systolic dysfunction
Loeper et al. 2016 [39]	Case-control	104 adults with MFS (45% female) and 111 adults with ns-TAAD (35% female)	148 healthy controls	2D echo Doppler TDI	Increased aortic stiffness index in MFS and ns-TAAD Reduced LV end-systolic elastance in MFS ventricular-vascular coupling index was abnormal in MFS No difference in LV stroke work in MFS
Winther et al. 2019 [30]	Case-control	69 adults (44% female)	20 age-matched controls	2D echo CMR	22% had reduced LV EF (≤55%) LV systolic dysfunction

BSA = Body surface area; CI = Confidence interval; CMR = Cardiac magnetic resonance imaging; EF = Ejection fraction; FS = Fractional shortening; LV = Left ventricle; LVEDD = Left ventricular end-diastolic diameter; LVESD = Left ventricular end-systolic diameter; MASS = Mitral valve, myopia, Aorta, Skin and Skeletal features; MFS = Marfan syndrome; MVP = Mitral valve prolapse; ns-TAAD = familial non-syndromal thoracic aortic aneurysm and dissection; TDI = Tissue Doppler imaging; * = Mean; ^ = Median; ( ) = Range.

**Table 3:** Summary of studies reporting end-stage heart failure necessitating heart transplantation in MFS.

Author	Type of Study	Number of Patients with MFS	Prior Aortic Surgery
Kesler et al. 1994 [42]	Survey	11	Not stated
Mullen et al. 1996 [43]	Case report	1	Yes
Varghese et al. 2006 [44]	Case report	1	Yes
Botta et al. 2006 [45]	Case report	1	Yes
Knosalla et al. 2007 [46]	Case series	10	Yes (100%)
Rajagopal et al. 2009 [47]	Case report	1	Yes
Audenaert et al. 2015 [49]	Case report	1	Yes
Rao et al. 2018 [40]	Case report	1	Yes
Ogawa et al. 2019 [41]	Case report	1	No

MFS = Marfan syndrome.

**Table 4:** Summary of studies assessing arrhythmia in MFS.

Author	Type of Study	Number of Patients with MFS	Controls	Assessment	Findings in MFS	
Chen et al. 1985 [81]	Follow-up: 5.7 years * (1–22)	24 children (63% female)	/	M-mode Resting ECG	33% presents at least 1 PVC on resting ECG 13% had VT QTc / QTUc prolongation was associated with ventricular arrhythmias Combination of abnormal repolarization and MVP associated with ventricular arrhythmias	
Savolainen et al. 1997 [80]	Case-control	45 adults (44% female)	45 healthy age and sex-matched controls	M-mode and 2D echo Ambulatory ECG	Higher median number of PACs than controls (12/24 h vs. 6/24 h; *p* < 0.05) Higher median number of PVCs than controls (17/24 h vs. 1/24 h; *p* < 0.001) More frequently repolarization abnormalities than controls Longer PQ- and QTc-intervals compared to controls 11% had NSVT°	
Yetman et al. 2003 [14]	Follow-up: 6 years ^ (1.6–24.5)	70 children and adults (51% female)	/	2D echo Resting ECG Ambulatory ECG	21% had ventricular ectopy (defined as >10 PVC/h) 6% had NSVT° 4% died from arrhythmias 16% had QTc prolongation and 60% had QTU prolongation Ventricular ectopy associated with LV size, MVP, and abnormalities of repolarization	
Hoffmann et al. 2012 [82]	Follow-up: 2.4 years ^ (2.1–2.7)	77 adults (52% female)	/	2D echo, Doppler and TDI Resting ECG and SAECG Ambulatory ECG	9% reached the composite endpoint (SCD, VT, VF or AS) 7% had VT 3% had SCD	
Aydin et al. 2013 [82]	Follow-up: 2.6 years *	80 children and adults (63% female)	/	M-mode and 2D echo Doppler Resting ECG Ambulatory ECG	91% had PVCs with 35% having >10 PVC/h 11% had NSVT° 8% had ventricular tachycardia events (SCD, VT, VF or AS) 4% had SCD Ventricular tachycardia events associated with NTproBNP and mutations in exons 24–32	
Schaeffer et al. 2015 [83]	Follow-up: 3.1 years *	102 adults (56% female)	/	2D echo Ambulatory ECG Heart rate turbulence	12% reached the primary endpoint (SCD, survived cardiac arrest, VT/VF and AS) 9% had VT 3% had SCD	
Arunamata et al. 2018 [85]	Case-control	45 children (44% female)	37 age, BSA, sex-matched controls	M-mode and 2D echo Resting ECG		Longer QTc intervals than controls
Mah et al. 2018 [79]	Cross-sectional	274 children and adults (38% female)	/	M-mode and 2D echo Ambulatory ECG		7% had ventricular ectopy (defined as >10 PVC/h) 5% had supraventricular ectopy (defined as >10 PAC/h) 1% had both supraventricular and ventricular ectopy None had VT or supraventricular tachycardia
Muiño Mosquera et al. 2020 [78]	Follow-up Case-control	86 children and adults (56% female)	40 age- and sex-matched controls	2D echo Resting ECG Ambulatory ECG NT-proBNP		Higher median number of PACs than controls (11/24 h vs. 2/24 h; *p* < 0.001) Higher median number of PVCs than controls (8/24 h vs. 0/24 h; *p* < 0.001) 23% had NSVT° Larger LVEDD and higher amount of VES were independently associated with NSVT°

AS = Arrhythmogenic syncope; LV = Left ventricle; MVP = Mitral valve prolapse; NSVT = Non-sustained ventricular tachycardia; PAC = Premature atrial complex; PVC = Premature ventricular complex; SAECG = Signal-averaged electrocardiography; SCD = Sudden cardiac death; TDI = Tissue Doppler imaging; TVP = Tricuspid valve prolapse; VF = Ventricular fibrillation; VT = Ventricular tachycardia; QTU = QT-interval measured from onset of QRS-complex to the end of the U-wave (if >50% of T-wave height); QTUc = QTU-interval corrected for heart rate; NSVT° defined as ≥3 consecutive PVCs at a heart rate >100 beats/min; * = Mean; ^ = Median; ( ) = Range.

## Abbreviations

BSA	Body surface area
CMR	Cardiac magnetic resonance imaging
DN	Dominant negative
EF	Ejection fraction
FBN1	Fibrillin-1
FS	Fractional shortening
HF	Heart failure
HI	Haploinsufficiency
LV	Left ventricular
LVEDD	Left ventricular end-diastolic diameter
LVESD	Left ventricular end-systolic diameter
MFS	Marfan syndrome
MVP	Mitral valve prolapse
NSVT	Non-sustained ventricular tachycardia
NTproBNP	N-terminal pro b-type natriuretic peptide
PAC	Premature atrial complex
PVC	Premature ventricular complex
RV	Right ventricular
SCD	Sudden cardiac death
TDI	Tissue Doppler imaging
TGF-β	Transforming growth factor beta
VF	Ventricular fibrillation
VT	Ventricular tachycardia
